# Supplementary material for: ER stress induced mitochondrial dysfunction drives Treg instability in coronary artery disease
Source: EMBO Mol Med. 2025 Oct 21;17(12):3250–74. doi: 10.1038/s44321-025-00322-3 (PMC12686412; doi:10.1038/s44321-025-00322-3)
Supplement: Supplementary file 1 — Appendix [file 44321_2025_322_MOESM1_ESM.pdf]

Appendix for ER stress induced mitochondrial dysfunction drives Treg instability in coronary artery disease

Table of contents

1) Appendix Figure S1: Page 1

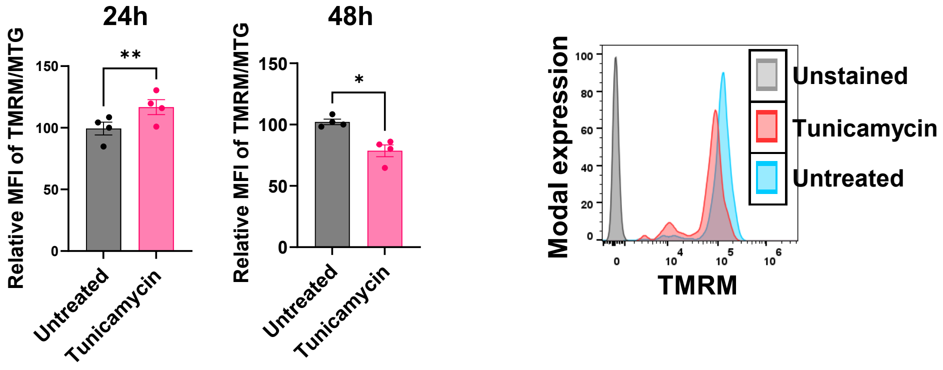

Appendix Figure S1: Human PBMCs were treated with tunicamycin for 24h and 48h and stained for TMRM and mito tracker green. The graph compares the ratio of MFI of TMRM to mito tracker (TMRM/MTG) in Tregs from untreated vs tunicamycin treated samples. Each dot represents a biological replicate from an independent human donor. n=4. Statistical comparisons were done using two-tailed T test. **= 0.0044 p value, *=0.0129
