# Supplementary figures and images for: ER stress induced mitochondrial dysfunction drives Treg instability in coronary artery disease
Source: EMBO Mol Med. 2025 Oct 21;17(12):3250–74. doi: 10.1038/s44321-025-00322-3 (PMC12686412; doi:10.1038/s44321-025-00322-3)

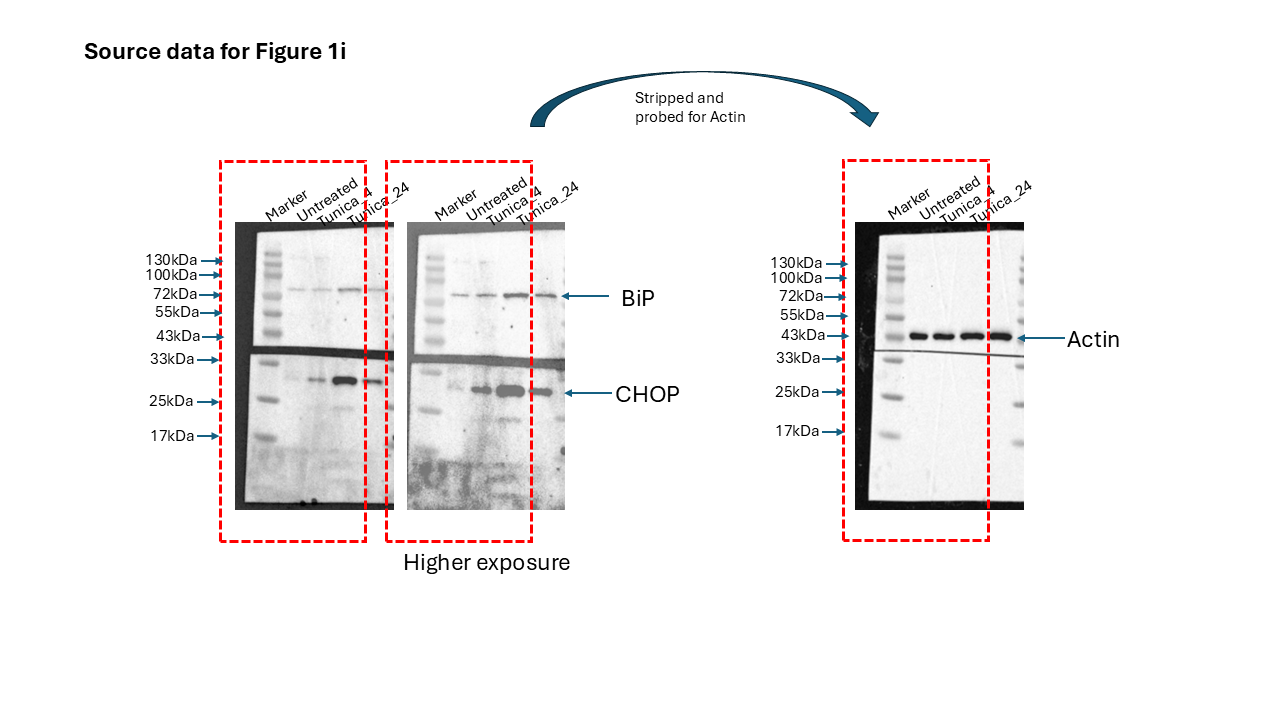

Supplement: Supplementary file 3 — Source data Fig. 1 [file 44321_2025_322_MOESM3_ESM.zip › EMM-2025-21765_Source data Figure 1/EMM-2025-21765_Source data Figure 1i/Western blot for Figure 1i.tif]

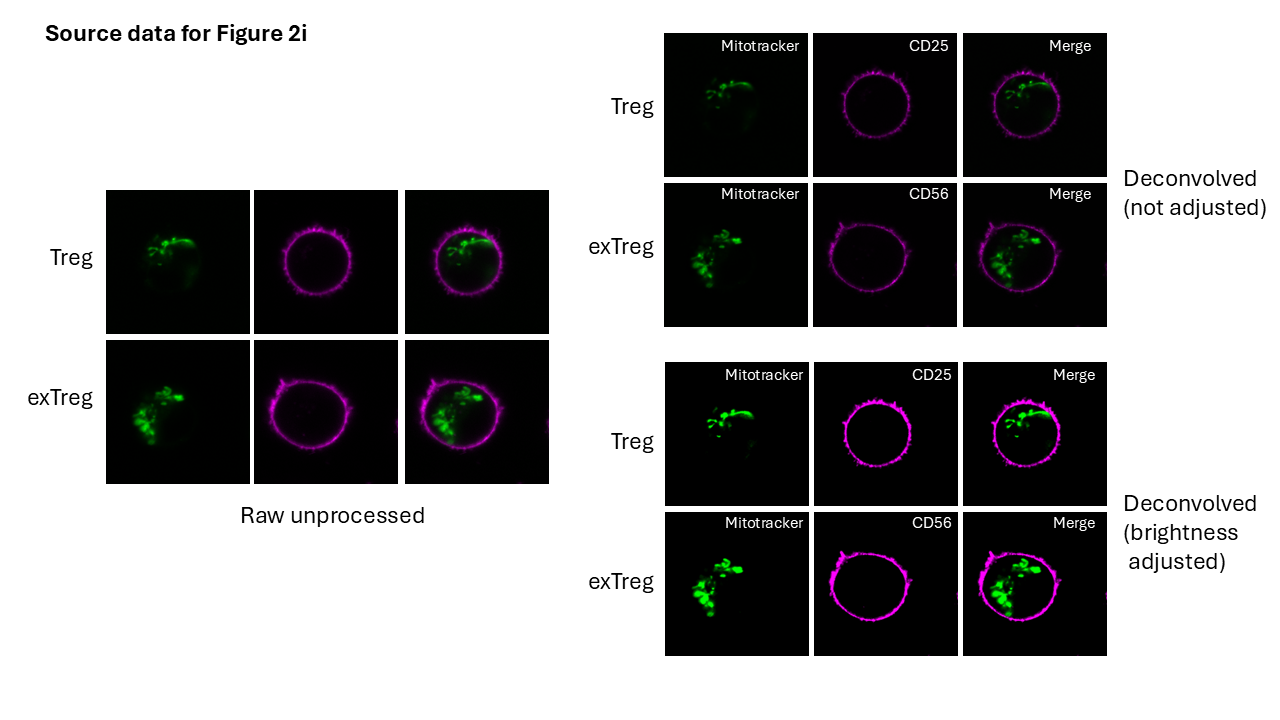

Supplement: Supplementary file 4 — Source data Fig. 2 [file 44321_2025_322_MOESM4_ESM.zip › EMM-2025-21765_Source data Figure 2/EMM-2025-21765_Source data Figure 2i/EMM-2025-21765-V3-Unprocessed_images_for_figure_2l.tif]

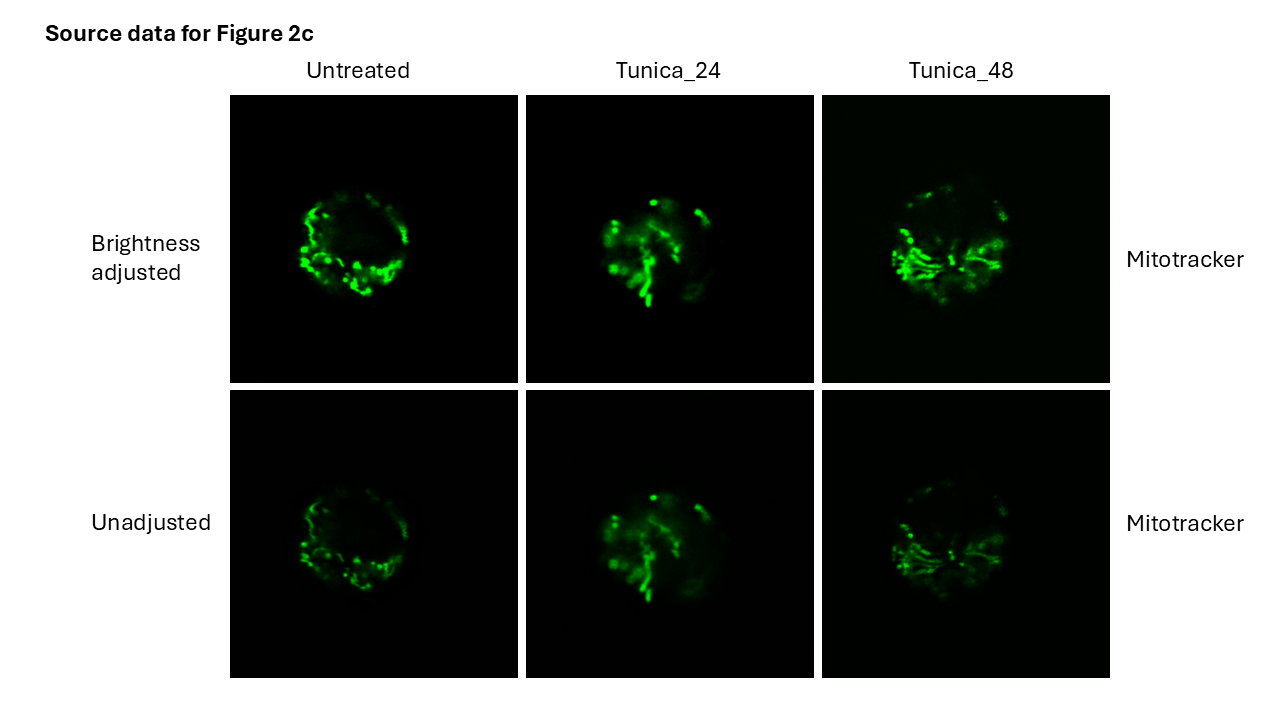

Supplement: Supplementary file 4 — Source data Fig. 2 [file 44321_2025_322_MOESM4_ESM.zip › EMM-2025-21765_Source data Figure 2/EMM-2025-21765_Source data Figure 2c/Microscopy images Figure 2c.tif]

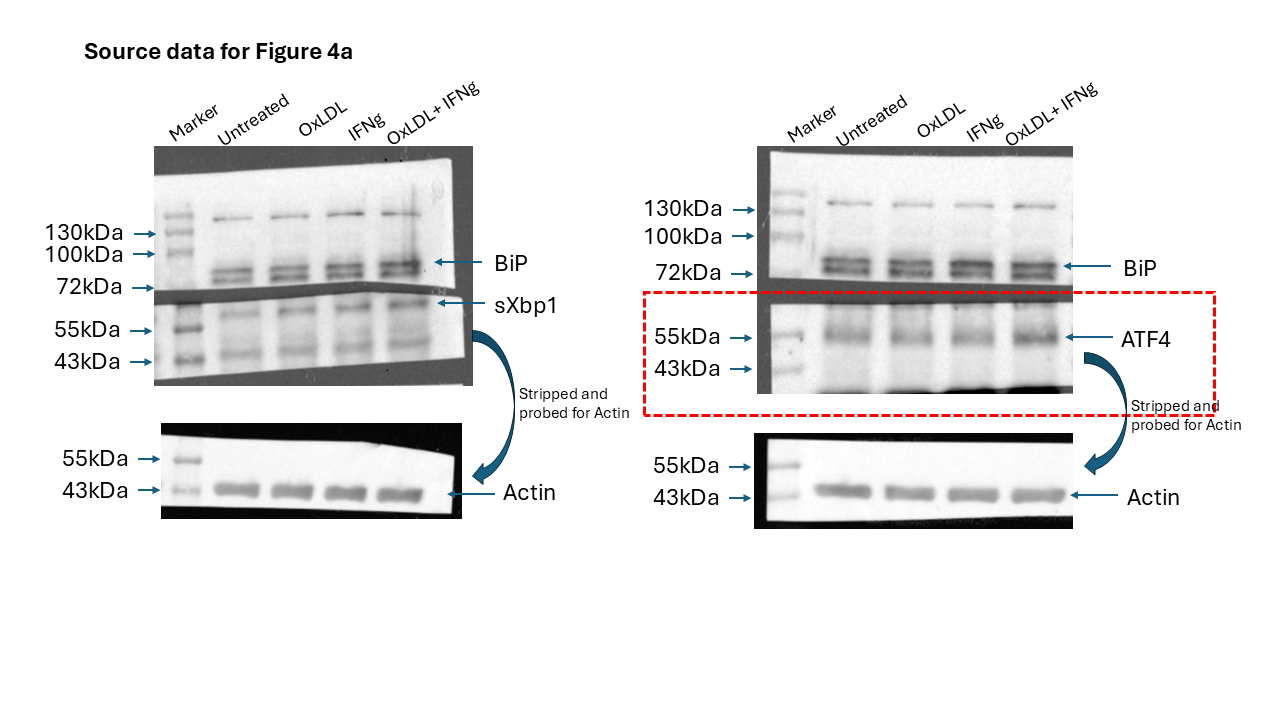

Supplement: Supplementary file 6 — Source data Fig. 4 [file 44321_2025_322_MOESM6_ESM.zip › EMM-2025-21765_Source data Figure 4/EMM-2025-21765_Source data Figure 4a/Western blot Figure 4a.tif]

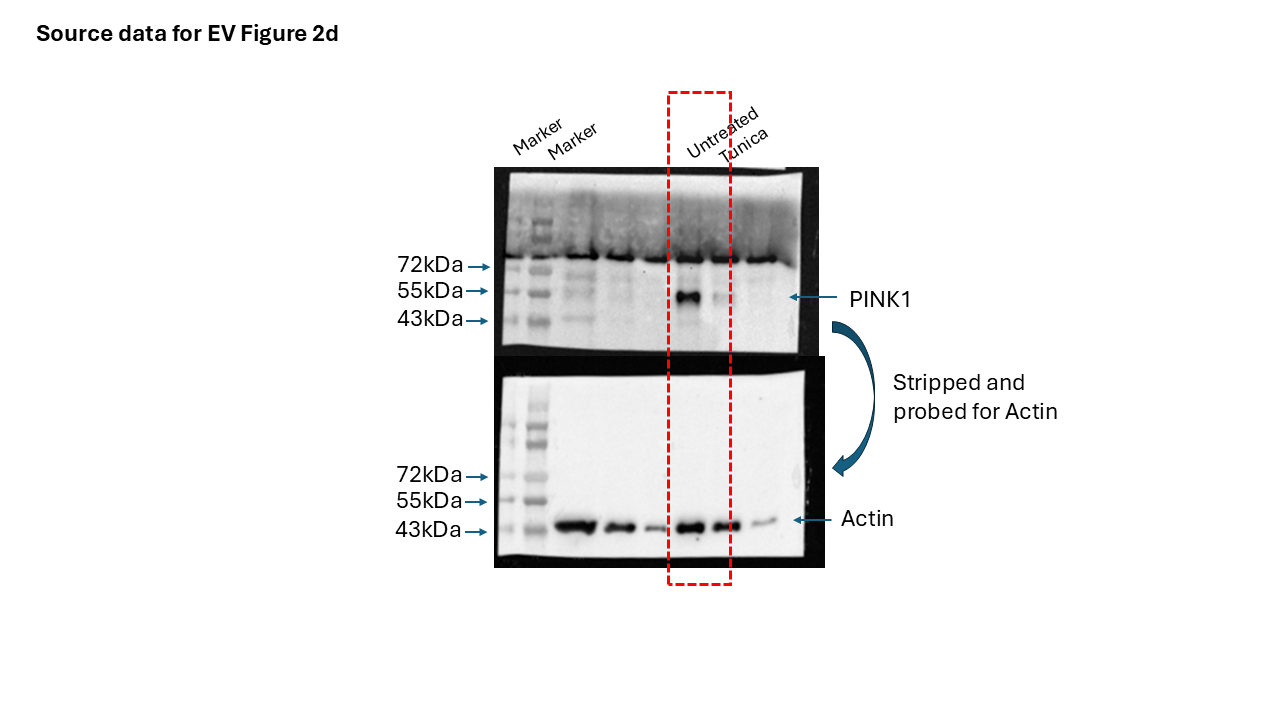

Supplement: Supplementary file 8 — Figure EV2 Source Data [file 44321_2025_322_MOESM8_ESM.zip › EMM-2025-21765_Source data EV Figure 2/EMM-2025-21765_source data EV Figure 2d/Western blot EV figure 2d.tif]
