# Supplementary material for: ER stress induced mitochondrial dysfunction drives Treg instability in coronary artery disease
Source: EMBO Mol Med. 2025 Oct 21;17(12):3250–74. doi: 10.1038/s44321-025-00322-3 (PMC12686412; doi:10.1038/s44321-025-00322-3)
Supplement: Supplementary file 10 — Expanded View Figures [file 44321_2025_322_MOESM10_ESM.pdf]

Expanded View Figures

Figure EV1. ER stress in human Tregs and exTregs.

(**A**) Normalized expression levels (transcripts per million) of ER-stress genes *XBP1, ATF6B* and *HSPA5* in human bulk transcriptomes from sorted human Tregs and exTregs. Horizontal bars represent the median. $n = 7$. (**B**) Gating strategy for identifying Tregs (CD3 + CD4 + CD8-CD25+CD127lo) and exTregs (CD3 + CD4 + CD8-CD25-CD56 + CD16 +) in human PBMC's. (**C–E**) Representative histograms for intracellular staining of IRE1 (**C**), PERK (**D**) and CHOP (**E**) by flow cytometry. Tregs (blue), exTregs (red) and isotype control (gray) are shown. The *y* axis was normalized to mode. (**F**) Representative histograms for intracellular staining of Proteostat by flow cytometry. Tregs (blue), Tregs + MG132 (purple) and unstained cells (gray) are shown. The *y* axis was normalized to mode. (**G**) Flow cytometry plots showing generation of iTregs by inducing CD3 + CD4 + CD45RA + CD45RA- Naïve T cells with ImmunoCult™ Human Treg Differentiation Supplement (containing TGFβ1 and all-trans retinoic acid) and Dynabeads human CD3/CD28 activator for 7 days (Left). Memory T cells (CD3 + CD4 + CD45RA-CD45RA +) were used as control (Right). (**H**) Representative flow plots showing frequency of CD25 + FOXP3+ Tregs in untreated vs Tunicamycin (72 h) treated PBMC's. (**I**) Bar graph showing frequency of CD25+CD127lo Tregs in PBMC's treated with Tunicamycin for 72 h. Untreated cells were used as control. Each dot represents a biological replicate from an independent human donor. $n = 6$. Representative flow plots are shown on the right. (**J**) natural Tregs (nTregs) or induced Tregs (iTregs) from same donors were treated with Tunicamycin for 24 h and relative MFI of FOXP3 was plotted against untreated controls from the respective donor. MFI of FOXP3 in untreated control from each donor was normalized to 100. $n = 3$. Each dot represents a biological replicate from an independent human donor. Statistical comparisons were done using two-tailed Mann–Whitney *U* test in (**A**) and using paired two-tailed *T* test in (**I**, **J**). Results are represented as mean ± SEM. Numerical *P* values are listed at the top of each bar graph. Source data are available online for this figure.

 © The Author(s)

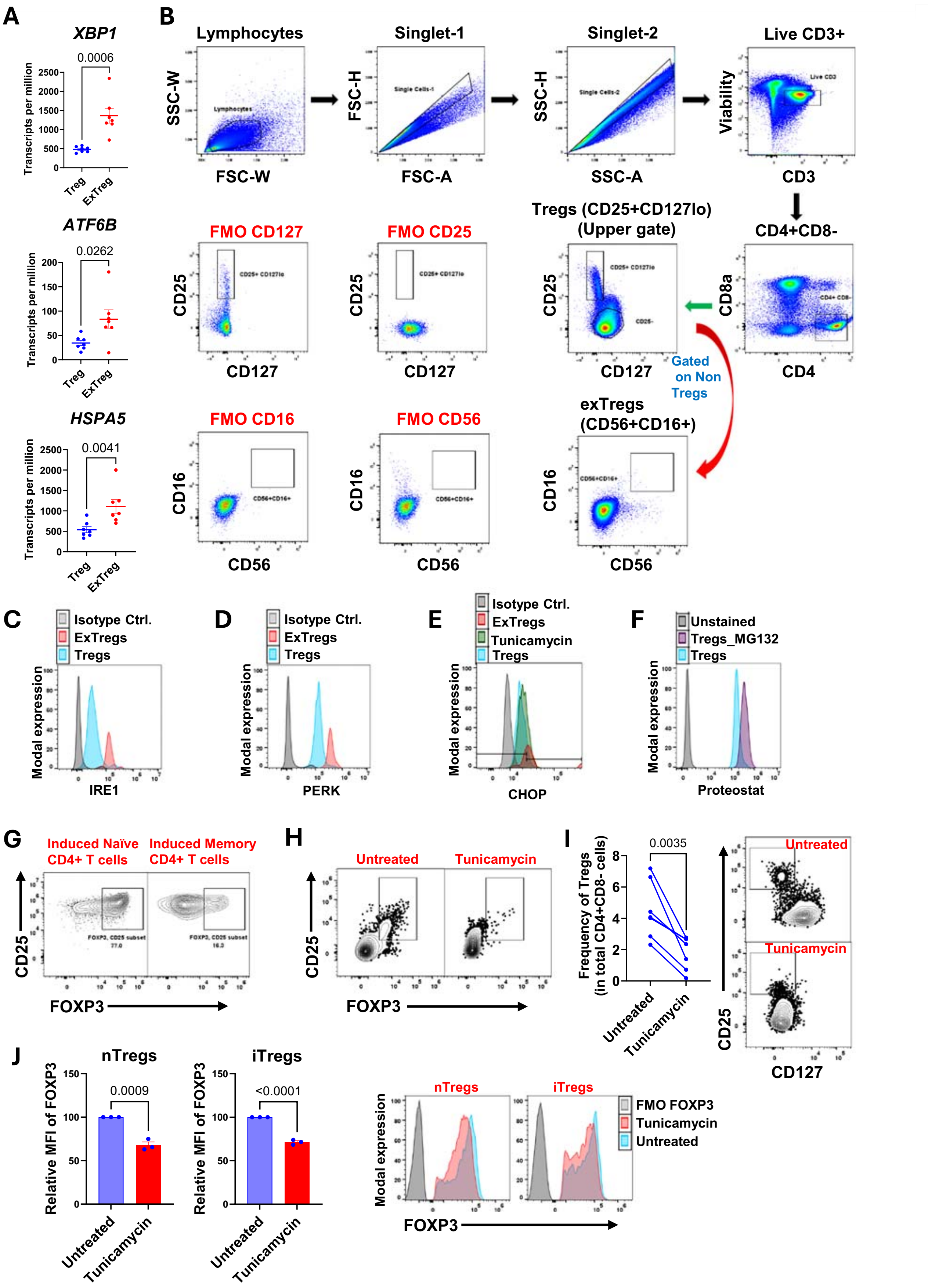
A
XBP1
0.0006
ATF6B
0.0262
HSPA5
0.0041
Transcripts per million
Treg
ExTreg
B
Lymphocytes
Singlet-1
Singlet-2
Live CD3+
SSC-W
FSC-W
FSC-H
FSC-A
SSC-H
SSC-A
Viability
CD3
CD4+CD8-
CD8a
CD4
Tregs (CD25+CD127lo)
(Upper gate)
FMO CD127
FMO CD25
CD25
CD127
Gated on Non Tregs
exTregs
(CD56+CD16+)
FMO CD16
FMO CD56
CD16
CD56
C
Isotype Ctrl.
ExTregs
Tregs
Modal expression
IRE1
D
PERK
E
Tunicamycin
CHOP
F
Unstained
Tregs_MG132
Proteostat
G
Induced Naïve CD4+ T cells
Induced Memory CD4+ T cells
FOXP3
H
Untreated
Tunicamycin
I
0.0035
Frequency of Tregs (in total CD4+CD8- cells)
J
nTregs
iTregs
0.0009
<0.0001
Relative MFI of FOXP3
FMO FOXP3

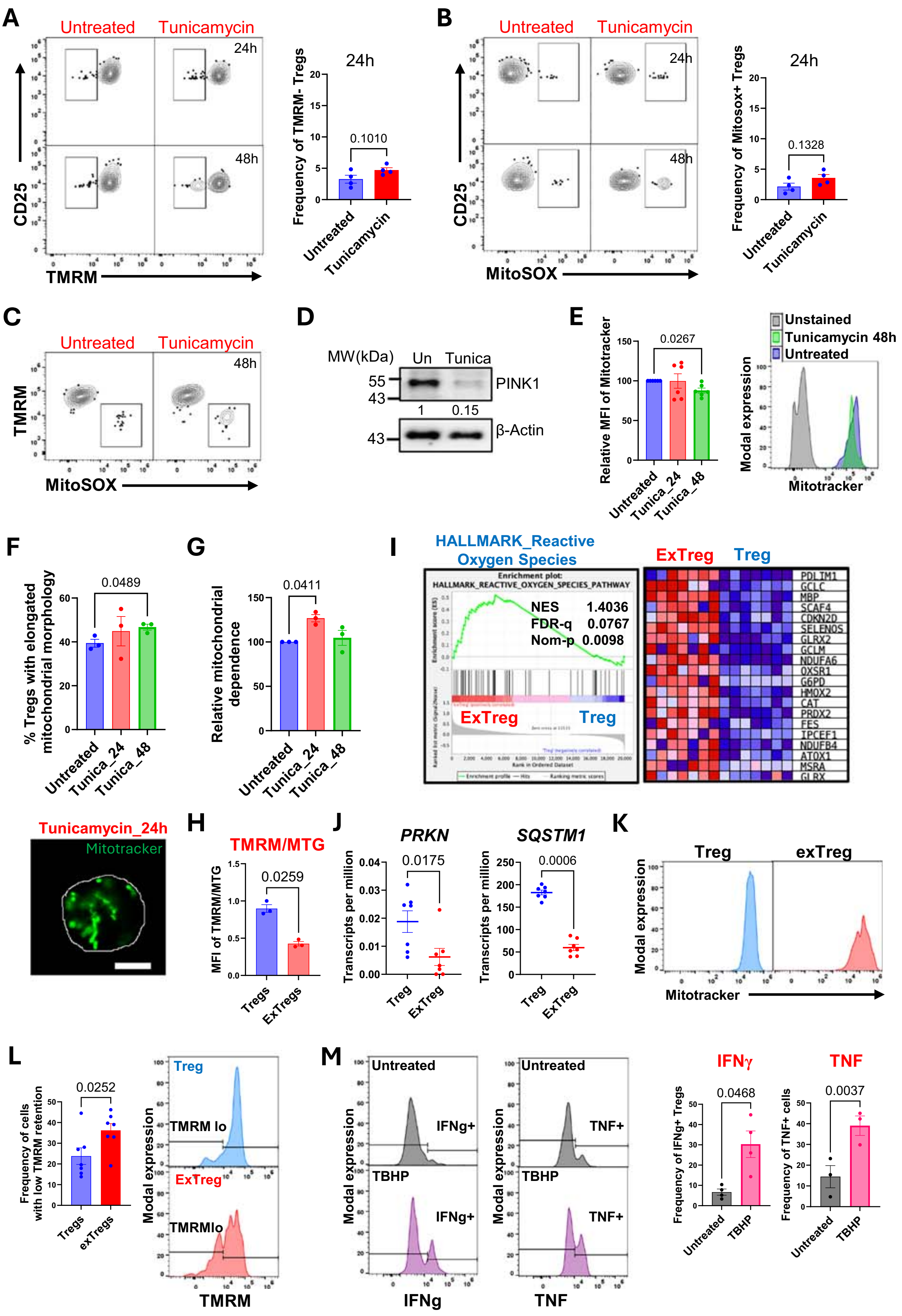
A
Untreated
Tunicamycin
24h
48h
CD25
TMRM
Frequency of TMRM- Tregs
0.1010
Untreated
Tunicamycin
B
MitoSOX
Frequency of Mitosox+ Tregs
0.1328
C
TMRM
D
MW(kDa)
Un
Tunica
55
43
PINK1
1
0.15
β-Actin
E
Relative MFI of Mitotracker
0.0267
Untreated
Tunica_24
Tunica_48
Unstained
Tunicamycin 48h
Modal expression
Mitotracker
F
% Tregs with elongated mitochondrial morphology
0.0489
G
Relative mitochondrial dependence
0.0411
I
HALLMARK_Reactive Oxygen Species
ExTreg
Treg
NES 1.4036
FDR-q 0.0767
Nom-p 0.0098
PDLIM1
GCLC
MBP
SCAF4
CDKN2D
SELENOS
GLRX2
GCLM
NDUFA6
OXSR1
G6PD
HMOX2
CAT
PRDX2
FES
IPCEF1
NDUFB4
ATOX1
MSRA
GLRX
Tunicamycin_24h
Mitotracker
H
TMRM/MTG
MFI of TMRM/MTG
0.0259
Tregs
ExTregs
J
PRKN
SQSTM1
Transcripts per million
0.0175
0.0006
Treg
ExTreg
K
exTreg
L
Frequency of cells with low TMRM retention
0.0252
exTregs
TMRM lo
TMRMlo
M
IFNg+
TBHP
TNF+
IFNg
TNF
IFNγ
Frequency of IFNg+ Tregs
0.0468
Frequency of TNF+ cells
0.0037

 © The Author(s)

Figure EV2. Tunicamycin and oxidative stress.

(**A**, **B**) Representative flow cytometry plots showing the frequency of TMRM− (**A**) or Mitosox+ (**B**) Tregs in PBMCs treated with tunicamycin for 24 h or 48 h. Untreated cells were used as control. Bar graph on the right of each plot shows the frequency of TMRM− (**A**) or Mitosox+ (**B**) Tregs after 24 h of tunicamycin treatment. Each dot represents a biological replicate from an independent human donor. $n = 4$. (**C**) Representative flow cytometry plots showing the frequency of Tregs that are TMRM- and Mitosox+ in untreated and tunicamycin treated samples. (**D**) In vitro generated iTregs were treated with tunicamycin for 24 h and the protein expression of PINK1 was analyzed by western blotting. Molecular weights (kDa) are indicated on the left of the blots. β Actin was used as loading control. Normalized levels of PINK1 to β Actin are indicated at the bottom of the blot. (**E**) Bar graph comparing the MFI of mitotracker in Tregs from PBMC's treated with tunicamycin for 24 h and 48 h. Untreated PBMC'S were used as control. Right, Representative histograms showing the fluorescence intensity of mitotracker in Tregs from untreated (blue) and tunicamycin treated (green) PBMC's. Unstained cells are shown in gray. The *y* axis was normalized to mode. (**F**) Tregs were cultured in vitro and treated with tunicamycin for 24 h and 48 h. Mitochondria were labeled with mitotracker green and visualized by live-cell confocal imaging. Untreated Tregs were used as control. Bar graph shows % of Tregs that showed tubular/elongated morphology. Each dot represents a biological replicate from an independent human donor. $n = 3$. Representative image for 24 h-tunicamycin treated samples are shown at the bottom. Scale bar, 5 µm. 15–30 cells were analyzed from each donor for each condition. (**G**) Human PBMC's were treated with tunicamycin for 24 h and 48 h and mitochondrial dependence of Tregs was determined using SCENITH. Bar graph shows relative mitochondrial dependence in tunicamycin treated cells compared to untreated control. Mitochondrial dependence in untreated control from each donor was set to 100. Each dot represents a biological replicate from an independent human donor. $n = 3$. (**H**) Human PBMCs were stained with TMRM and Mitotracker green and analyzed by flow cytometry. MFI of TMRM/MTG in Tregs from each donor was calculated and MFI of TMRM/MTG for exTregs from respective donors was plotted against it. Each dot represents a biological replicate from an independent human donor. $n = 3$. (**I**) GSEA plots showing enrichment of Hallmark gene signature for Reactive Oxygen Species (M5938 in mSigDB) within exTreg and Treg ($n = 7$) transcriptomes. Normalized enrichment score (NES), FDR q and nominal p values are indicated. Heatmap for top 10 enriched genes in exTregs vs Tregs is shown at bottom. Color scale in the heatmap is based on GSEA row minimum (blue) to row maximum (red). (**J**) Normalized expression levels (transcripts per million) of mitophagy genes *PRKN* and *SQSTM1* in human bulk transcriptomes from sorted human Tregs and exTregs. Horizontal bars represent the median. $n = 7$. (**K**) Representative flow cytometry plots showing mitotracker staining in Tregs (blue) and exTregs (red). *y* axis was normalized to mode. (**L**) Human PBMC's were stained with TMRM and analyzed by flow cytometry. Bar graph on left depicts the frequency of Tregs and exTregs that show low TMRM retention. Representative flow cytometry plots for Tregs (blue) and exTregs (red) are shown on the right. y axis was normalized to mode. (**M**) Human PBMC's were treated with TBHP and frequency of cells that express IFN γ or TNF was analyzed by flow cytometry. Untreated cells were used as control. Representative flow cytometry plots for each cytokine (untreated in gray and TBHP treated in purple) are shown on the left. Bar graphs comparing the frequency are shown on the right. $n = 4$ for IFN γ and $n = 3$ for TNF. Each dot represents a biological replicate from an independent human donor. Statistical comparisons were done using paired two-tailed T test in (**A**, **B**, **H**, **L**, **M**), one-way ANOVA with Tukey's multiple comparisons in (**E–G**) and two-tailed Mann–Whitney *U* test in (**J**). Results are represented as mean ± SEM. Numerical *P* values are listed at the top of each bar graph. Source data are available online for this figure.

© The Author(s)

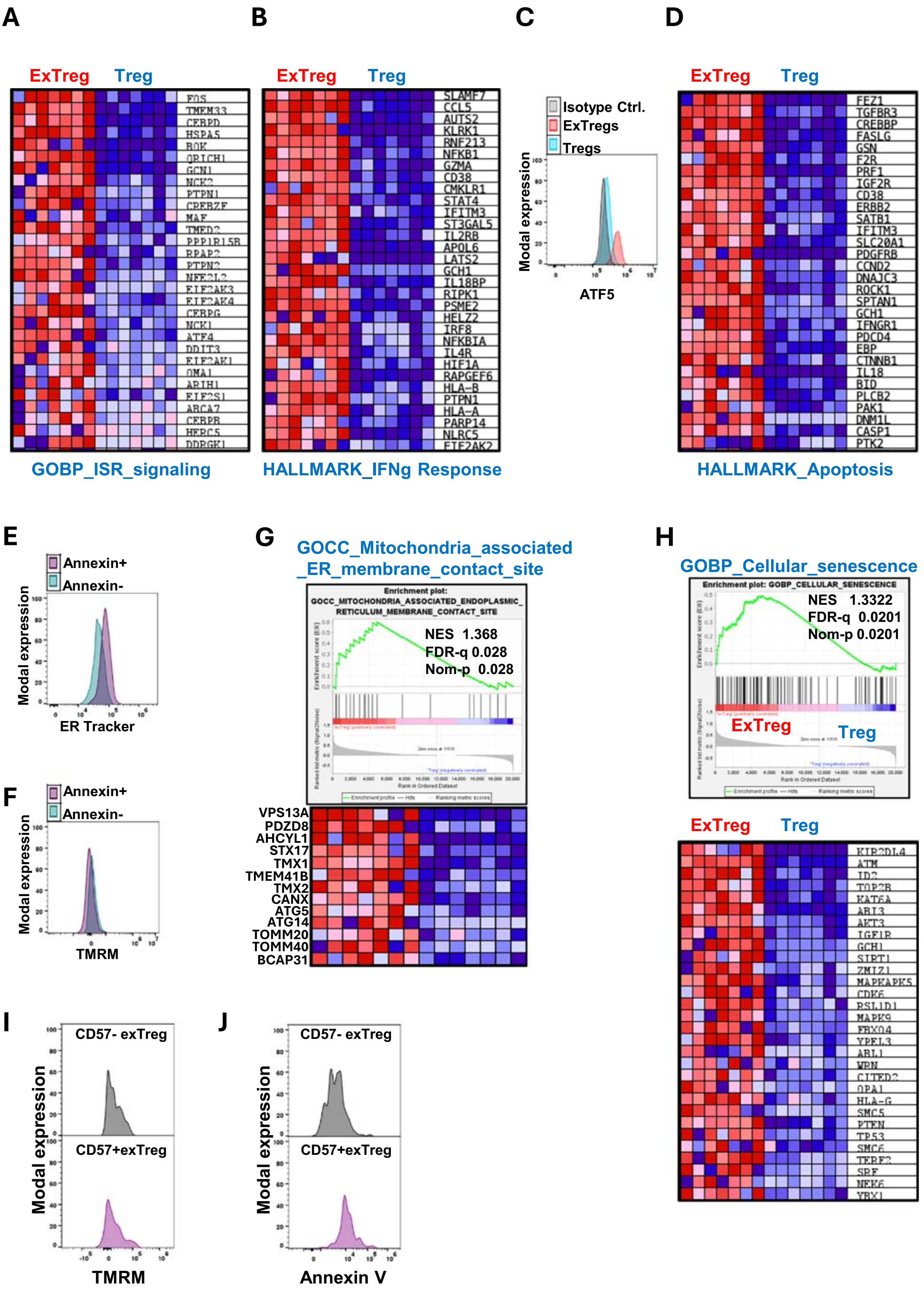
A
B
C
D
ExTreg
Treg
GOBP_ISR_signaling
HALLMARK_IFNg Response
Isotype Ctrl.
ExTregs
Tregs
Modal expression
ATF5
HALLMARK_Apoptosis
E
Annexin+
Annexin-
ER Tracker
F
TMRM
G
GOCC_Mitochondria_associated_ER_membrane_contact_site
NES 1.368
FDR-q 0.028
Nom-p 0.028
VPS13A
PDZD8
AHCYL1
STX17
TMX1
TMEM41B
TMX2
CANX
ATG5
ATG14
TOMM20
TOMM40
BCAP31
H
GOBP_Cellular_senescence
NES 1.3322
FDR-q 0.0201
Nom-p 0.0201
I
J
CD57- exTreg
CD57+exTreg
Annexin V

Figure EV3. Gene set enrichment for integrated stress response and interferon gamma response.

(**A**, **B**) Heatmaps showing top 30 enriched genes in exTreg vs Treg transcriptomes as revealed by GSEA for I\integrated stress response signaling (**A**) and interferon gamma response (**B**). Color scale in the heat map is based on GSEA row minimum (blue) to row maximum (red). (**C**) Representative flow cytometry plot for ATF5 staining in Tregs (blue), exTregs (red) and isotype control (gray). Y axis was normalized to mode. (**D**) Heatmap showing top 30 enriched genes in exTreg vs Treg transcriptomes as revealed by GSEA for Apoptosis. Color scale in the heat map is based on GSEA row minimum (blue) to row maximum (red). (**E**, **F**) Representative flow cytometry plots comparing ER tracker (**E**) and TMRM (**F**) staining in Annexin+ (purple) and Annexin- (turquoise) exTregs. *Y* axis was normalized to mode. (**G**, **H**) GSEA plots showing enrichment of gene signature for mitochondria-associated ER membrane contact sites (GO:0044233 in mSigDB) in (**G**) and cellular senescence (GO:0090398) in (**H**) within paired exTreg and Treg ($n = 7$) transcriptomes. Normalized enrichment score (NES), FDR q and Nominal *P* values are indicated. Top enriched genes in exTregs are shown at the bottom. Color scale in the heat map is based on GSEA row minimum (blue) to row maximum (red). (**I**, **J**) Representative flow cytometry plots comparing TMRM (**I**) and Annexin V (**J**) staining in CD57− (gray) and CD57+ (purple) exTregs. Y axis was normalized to mode.

© The Author(s)

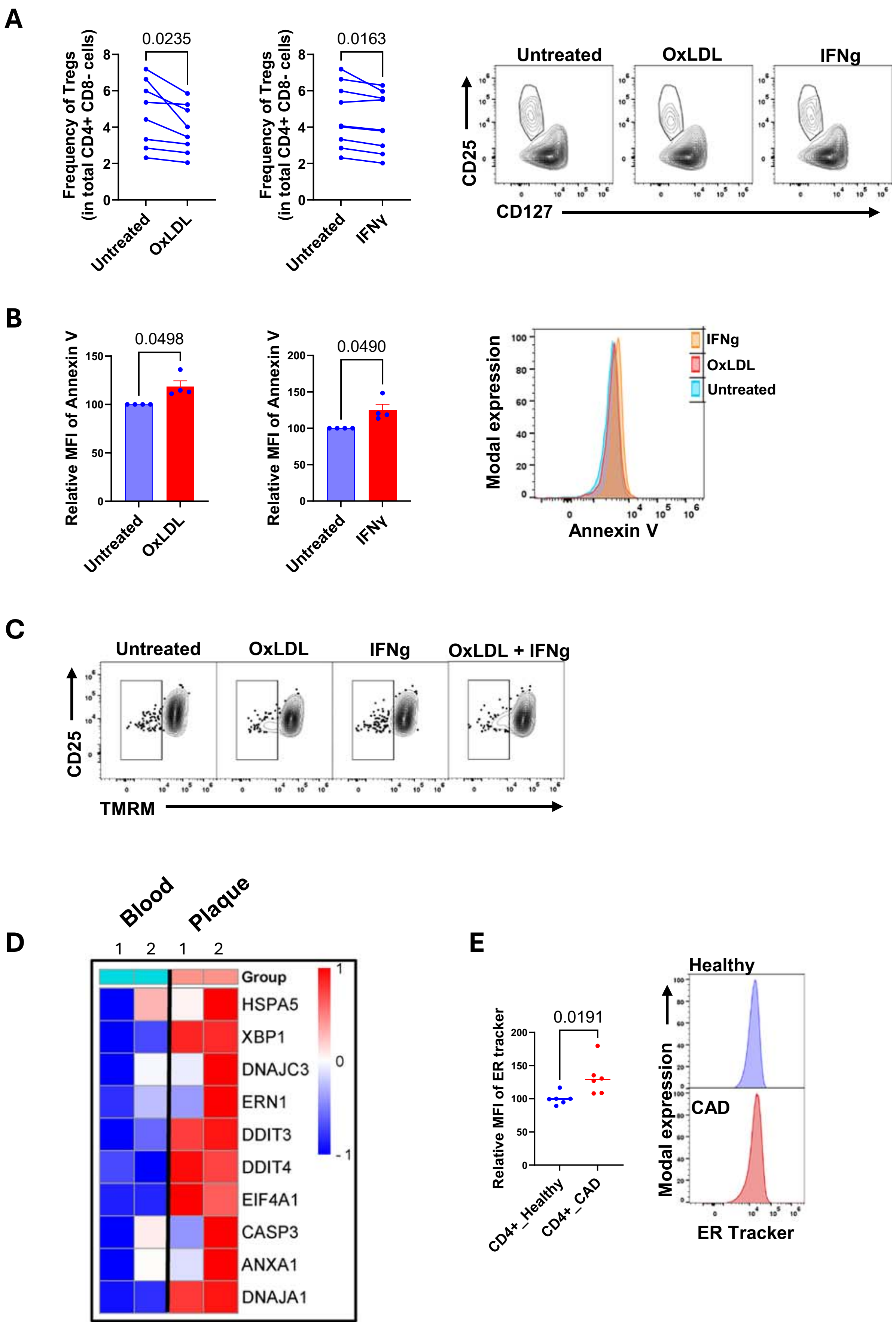
A
Frequency of Tregs (in total CD4+ CD8- cells)
0.0235
Untreated
OxLDL
0.0163
IFNγ
Untreated
OxLDL
IFNg
CD25
CD127
B
Relative MFI of Annexin V
0.0498
0.0490
Modal expression
IFNg
OxLDL
Untreated
Annexin V
C
Untreated
OxLDL
IFNg
OxLDL + IFNg
CD25
TMRM
D
Blood
Plaque
Group
HSPA5
XBP1
DNAJC3
ERN1
DDIT3
DDIT4
EIF4A1
CASP3
ANXA1
DNAJA1
E
Relative MFI of ER tracker
0.0191
CD4+_Healthy
CD4+_CAD
Healthy
CAD
Modal expression
ER Tracker

 © The Author(s)

Figure EV4. oxLDL and IFNγ treatment.

(**A**) Human PBMCs were treated with oxLDL or IFN γ and frequency of CD25+ CD127lo Tregs was compared by flow cytometry. Untreated cells were used as control. Each dot represents biological replicate from an independent human donor. $n = 8$ for oxLDL and $n = 9$ for IFN γ. Representative flow cytometry plots are shown on the right. (**B**) MFI of Annexin V in Tregs from oxLDL or IFN γ treated PBMC's. Untreated cells were used as control. MFI of Annexin V in untreated cells was normalized to 100 and relative MFI of treated samples was plotted against it. $n = 4$. Each dot represents biological replicate from an independent human donor. Representative flow cytometry plots are shown on the right. (**C**) Representative flow cytometry plots for TMRM staining in Tregs from PBMC's treated with oxLDL, IFN γ or oxLDL + IFN γ. (**D**) Heatmap comparing the expression of different ER stress and apoptosis related genes in Tregs circulating in blood or residing in plaque of CAD patients (GSE196943). Blood and plaque cells are matched by the same patients. (**E**) MFI of ER tracker in CD4 + CD8- T cells from healthy and CAD patients. $n = 6$. Each dot represents an independent donor. Representative histograms for healthy (blue) and CAD (red) are shown on the right. Horizontal bars depict the median. Statistical comparisons were done using paired two-tailed *T* test in (**A**, **B**) and unpaired two-tailed *T* test in (**E**). Results are represented as mean ± SEM. Numerical *P* values are listed at the top of each bar graph. Source data are available online for this figure.

© The Author(s)
